# IntelliCage protocols using incentive-disincentive dual motivation work with male as well as female mice and reveal sex differences in motivated behavior

**DOI:** 10.3389/fnbeh.2026.1833013

**Published:** 2026-05-26

**Authors:** Laurine Roos, Gian Rieder, Irmgard Amrein, David P. Wolfer

**Affiliations:** 1 Division of Functional Neuroanatomy, Institute of Anatomy, University of Zurich, Zurich, Switzerland; 2 Institute of Human Movement Sciences and Sport, Department of Health Sciences and Technology, ETH Zurich, Zurich, Switzerland

**Keywords:** animal welfare, appetitive stimulus, aversive stimulus, dual motivation protocol, group-housed rodents, IntelliCage system, spatial learning, sex differences

## Abstract

**Introduction:**

In the IntelliCage system, spontaneous behavior and cognitive abilities of mice can be assessed automatically in a social homecage context and without handling by human experimenters. We have recently shown that female mice can be motivated to learn complex spatial tasks with refined IntelliCage protocols that lack water restriction and thereby eliminate the risk of water deprivation in poor learners. These protocols create a dual motivation by combining incentive and disincentive taste stimuli. The mice learn to gain access to saccharin sweetened water while water rendered bitter by quinine remains permanently available.

**Methods:**

Here, we used a total of 64 C57BL/6J mice to assess males and females in dual motivation protocols versus single motivation protocols using only saccharin as incentive stimulus.

**Results:**

We found that in dual motivation protocols male mice learned at least as well as females provided that the location of target corners was stable over time. When targets shifted frequently and in response to the choices of the animals, male mice lost motivation and learned poorly. Based on lick counts, we did not find evidence of impaired liquid intake despite the addition of a disincentive stimulus.

**Discussion:**

Our study demonstrates that by using dual motivation protocols, it is possible to create spatial learning tasks for IntelliCage which do not enforce learning by water restriction and which can be learned by mice of both sexes. Furthermore, our results reveal an interesting sex difference in motivated behavior which is not apparent in protocols that enforce learning by water restriction. We hypothesize that this sex difference may be related to the socio-spatial organization of male mouse groups in the IntelliCage.

## Introduction

Because of increasing concerns regarding animal welfare and poor reproducibility of conventional behavioral tests (Crabbe et al., 1999; Chesler et al., 2002; van der Staay and Steckler, 2002; Wahlsten et al., 2003; Voikar et al., 2005; d’Isa and Gerlai, 2022; Nigri et al., 2022) alternative approaches based on continuous monitoring of mice in their homecage have become increasingly popular. They enable the assessment of behavior over longer periods across the full circadian cycle in familiar, low-stress environments, and offer more comprehensive and ecologically valid insights into behavioral patterns (Voikar and Gaburro, 2020; Kahnau et al., 2023; Teatime-Cost-Action-CA20135-Welfare-Group, 2025; Jurek et al., 2026).

While most homecage-based systems require isolation of the animals, the IntelliCage system permits the behavioral assessment of mice in their familiar social homecage context. To date, over 170 publications in PubMed mention the use of IntelliCage. More than 40 protocols have been developed by around 80 research groups worldwide (Lipp, 2005; Kiryk et al., 2020; Lipp et al., 2024). In typical IntelliCage tasks, mice respond and learn to obtain plain or sweetened water as rewards, with incorrect responses blocking access to water (Onishchenko et al., 2007; Endo et al., 2011; Kobayashi et al., 2013; Masuda et al., 2016; Kiryk et al., 2020; Lipp et al., 2024). In some studies, access to liquid is additionally limited to short drinking sessions (Codita et al., 2010; Krackow et al., 2010; Weyer et al., 2011; Voikar et al., 2018; Mehr et al., 2020). While these designs yield a strong motivational drive to learn even difficult tasks, they pose challenges for poor learners which are at risk of dehydration. In addition, this may result in the early removal of animals from the study, leading to a reduced sample size and diminished statistical power.

To further refine the IntelliCage system and to better align it with the 3R principles, our group attempted to develop new IntelliCage protocols for spatial learning in which plain water remains freely available but learning the task rule gives the mice access to sweetened water as additional rewards and learning incentive. However, this approach failed to motivate female mice to learn difficult hippocampus-dependent tasks (Bramati et al., 2023). Male mice, despite being similarly attracted to sweet rewards in absence of challenge, lost motivation to engage in learning tasks even more quickly than females (Nigri et al., 2024). These observations led us to design dual motivation protocols for IntelliCage in which the mice still work and learn for sweet rewards, but with a disincentive motivational component added by rendering the permanently available water less attractive with bitter tasting quinine (Ma et al., 2023). In female mice, these protocols strongly improved motivation and performance even in difficult tasks, without the added disincentive having an obvious detrimental impact on liquid intake, health status or body weight of the animals.

It is now widely recognized that experimental studies can only be fully valid if study subjects of both sexes are included, not only in clinical (Witt et al., 2026) but also in preclinical studies involving animal models (Justice, 2024). Accordingly, many funding agencies demand study designs which include experimental animals of both sexes. Therefore, the present study tested whether our dual motivation protocols (Ma et al., 2023) for IntelliCage are similarly effective in motivating task engagement and allow good performance in mice of both sexes. Using a battery of tasks, we tested whether motivation and performance would be maintained across a range of increasingly difficult learning rules. We present evidence that dual motivation based on the combination of incentive and disincentive taste stimuli also motivates male mice to engage in complex hippocampus-dependent tasks and to reach good performance – provided that location of target corners remains stable. In tasks involving frequently shifting targets, male mice remain less willing than females to adopt the learning rule despite dual motivation.

## Materials and methods

Animal experiments were performed at the Institute of Anatomy, University of Zurich. The procedures received approval from the veterinary office of the Canton of Zurich (license ZH060/2021) and were supervised by the Office for Animal Welfare and 3R of the University of Zurich.

### Breeding and preparation of animals

To breed the 96 C57BL/6J mice (48 males and 48 females) used in this study, we ordered 15 pregnant female mice (Janvier Labs, France). Their offspring were weaned at 21 days of age and housed in six male and six female groups of eight in standard type III cages. Selection and assignment to cages was randomized to avoid litter effects by co-grouping of siblings. The mice were kept on a 12-h inverted light-dark cycle, with the lights turned on from 8:00 p.m. to 8:00 a.m. Temperature in the animal facility was 23 °C, humidity 28%–36%. Standard food pellets and water were available ad libitum. Body weight was measured at regular intervals to monitor the health status of the mice. At the age of 2.5 months, 7 days before introduction to the IntelliCage, RFID transponders (Planet-ID GmbH/Datamars, Germany) were implanted subcutaneously in the dorso-cervical area of the mice under isoflurane anesthesia. By the end of the study, the mice had reached the age of 5.5 months.

### The IntelliCage system

The IntelliCage system (TSE Systems, Bad Homburg, Germany) is a fully automated system that allows to study the behavior and cognitive abilities of up to 16 mice while group housed in a familiar homecage environment (Ma et al., 2023; Lipp et al., 2024). Our setup consists of two polycarbonate cages. The main type 2000 cage measuring 612 × 435 × 216 mm (Tecniplast SA, Buguggiate, Italy) is connected by a tube to a standard type III extension cage with dimensions of 425 × 266 × 155 mm to provide additional living space. The main cage contains four central triangular mouse shelters (ZOONLAB GmbH) and an U-shaped metal feeder providing standard food pellets ad libitum. A metal frame inside the main cage holds four identical operant corners, each featuring two motorized doors: one on the long side of the cage, referred to as the “task side” door in this study, and one on the short side of the cage, referred to as the “joker side” door in this study. A bottle is placed behind each door, accessible for drinking upon opening of the door.

Various sensors detect the behavior of RFID-transpondered animals. An RFID antenna identifies animals as they enter a corner and a temperature-sensitive presence sensor detects start, duration, and end of each visit to the corner. An infrared (IR) light beam positioned in front of the doors regulating access to the drinking spouts detects nose pokes. A lickometer measures number and duration of tongue contacts with the drinking nozzle. The learning protocols are configured using IntelliCage’s Designer software (IntelliCage Plus, TSE Systems, Bad Homburg, Germany). The Controller software then monitors and records visits, nose pokes and licks in real time, and uses them to trigger door actions following the selected protocol.

### Dual and single motivation protocols

To induce learning, we used a dual motivation protocol using both incentive and disincentive stimuli (Ma et al., 2023). The incentive stimulus was a 0.5% solution of saccharin sodium salt hydrate (Sigma-Aldrich S1002), while the disincentive stimulus was a 0.3 mM solution of quinine monohydrochloride dihydrate (Acros Organics A0420352). The saccharin and quinine concentrations were used successfully in the previous study (Ma et al., 2023) and were originally determined in pilot experiments to achieve an optimal balance between behavioral effectiveness and welfare-compatibility.

**FIGURE 1:**
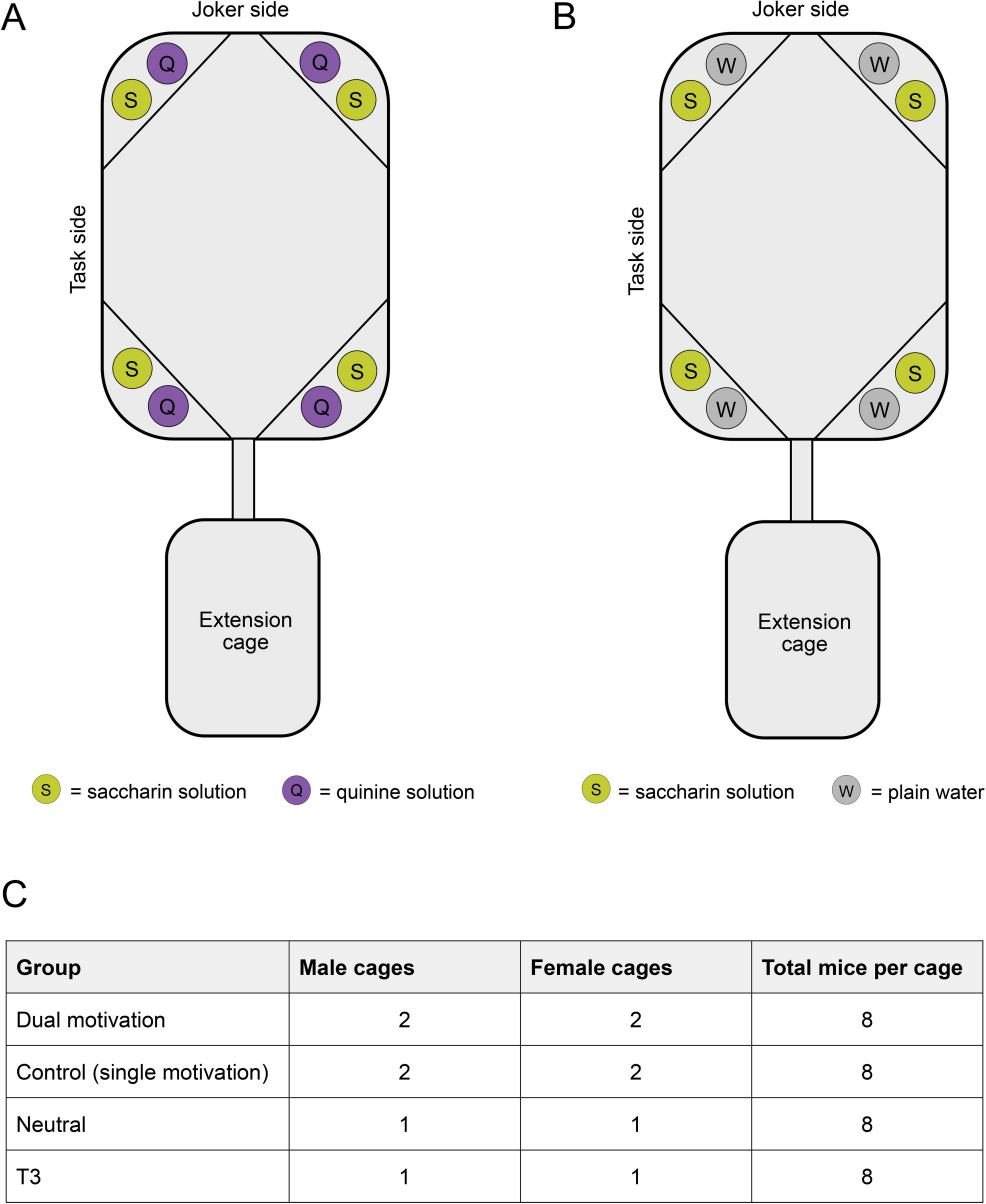
IntelliCage design. **(A)** In the dual motivation group, task side bottles contained sweet saccharin solution, while joker side bottles contained bitter quinine solution. **(B)** In the control group, task side bottles contained sweet saccharin solution, and joker side bottles contained plain water. **(C)** Table showing the distribution of experimental groups across IntelliCages.

In the dual motivation group, bottles on the task side were filled with the sweet and rewarding saccharin solution, and bottles on the joker side contained the bitter quinine solution (dual motivation protocol, Figure 1A). For the control group, the task side bottles also contained the saccharin solution, but the joker side bottles were filled with plain water (single motivation protocol, Figure 1B).

**FIGURE 2:**
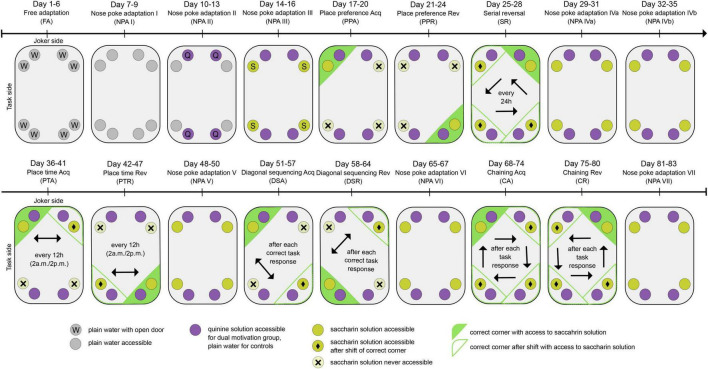
Description and timeline of adaptation, learning and recovery phases in the IntelliCage system. Free adaptation: Mice were habituated to the IntelliCage environment, with all eight doors open, allowing unrestricted access to plain water (W). Nose poke adaptation I: All drinking bottles still contained water. During this and all subsequent phases, the joker door opened automatically at the start of each corner visit for 3 s, providing access to the drinking bottle. The task door also provided access to the drinking bottle but opened for 3 s only following a nose poke. This mode of task door operation was maintained during all subsequent adaptation phases and in correct corners during all learning tasks. Nose poke adaptation II: In the dual motivation group, joker side bottles were filled for the first time with quinine solution (Q), while the control group continued to receive plain water at joker sides. Nose poke adaptation III: Saccharin solution was introduced to the task side bottles (S). During learning tasks, at any time only one of four corners was correct (green) and provided access to saccharin. Place preference acquisition and reversal: Each mouse was assigned one permanent correct corner. Joker-side drinking bottles were accessible at the start of every visit at all corners. Task-side saccharin bottles were accessible only in the correct corner, and only after a nose poke. During reversal, the correct corner shifted diagonally. Serial reversal: The correct corner changed every 24 h following a complex pseudo-random pattern. Place time acquisition and reversal: Each mouse was assigned an initial correct corner, alternating between two adjacent corners depending on the time of day (switches at 2 a.m. and 2 p.m.). Reversal involved using the opposite two corners with a reversed shift direction. Diagonal sequencing acquisition and reversal: The correct corner changed diagonally after each correct task response. During reversal, the remaining two corners were used under the same rule. Chaining acquisition and reversal: The correct corner followed a clockwise or counterclockwise sequence, changing after each task response. The sequence direction was reversed during the reversal phase. Recovery phases: Nose poke adaptation protocols were repeated two times after the first block of learning tasks (NPA IVa+b) and once after the following blocks (NPA V–VII) to reset task performance prior to the next learning task.

During the first 16 days in the IntelliCage, mice were habituated to the new environment and to the stimuli (Figure 2). Habituation began with free adaptation (FA) during which all doors were always open, providing free access to bottles containing plain water. During nose poke adaptation I (NPA I), the mice were habituated to the operation of doors on joker and task sides with all bottles still containing plain water. Quinine was added for the dual motivation group to joker sides during nose poke adaptation II (NPA II), while saccharin was introduced to task sides for both groups during nose poke adaptation III (NPA III). Following the adaptation phases, mice were subjected to a series of learning tasks that became increasingly difficult over 83 days, as the correct corner either changed periodically or shifted in different patterns depending on responses (for a detailed explanation of the learning protocols, refer to Figure 2). The start of each corner visit automatically triggered the opening of the joker door for 3 s, allowing the mice access to either the quinine solution or plain water. However, the task door opened for 3 s only following a nose poke in the corner that was correct according to the current spatial task rule, granting the mice access to the saccharin solution. Initial correct corners of the acquisition phase of each learning task were randomized across animals in a manner balancing targets across the four corners of each cage. For each mouse, the spontaneously least and most preferred corner of the preceding NPA phase were not used to minimize bias on the baseline. Clock-wise and anticlock-wise shift directions in the chaining task were randomized across animals in a manner assuring an equal number of clock- and anticlock-wise running animals in each cage. To reset task performance between different learning tasks, recovery phases with the protocol of nose poke adaptation III were interspersed (NPA IVa+b, V–VII).

### Experimental groups

To ensure balanced distribution of males and females to experimental groups, each of the 12 housing groups established at weaning was randomly assigned to one of the experimental groups and transferred as a whole to an IntelliCage, in a manner resulting in the following balanced arrangement (Figure 1C): four IntelliCages (32 mice, *M* = 16 in 2 cages, F = 16 in 2 cages) formed the dual motivation group, four IntelliCages (32 mice, *M* = 16 in 2 cages, F = 16 in 2 cages) the control (single motivation) group. Because learning corners can hold only two drinking bottles, containing either plain water or water with quinine at joker sides, we had to keep dual motivation groups and controls in separate cages. Two additional IntelliCages (16 mice, *M* = 8 in 1 cage, F = 8 in 1 cage) formed a neutral group remaining under the free adaptation protocol with plain water throughout the experiment. Finally, two housing groups (16 mice, *M* = 8 in 1 cage, F = 8 in 1 cage) formed the baseline T3 group. They were not transferred to IntelliCages but kept in the experimental room in their standard type III cages. The neutral and T3 groups were welfare controls used as reference for assessing licking, health status, and changes in body weight.

### Behavioral parameters

The response task ratio R was used as a measure of the preference for responding to task sides, reflecting the motivation of the mice to engage in the learning task (Ma et al., 2023). The following formula was used to calculate R:


$$R=\frac{2+2\times \mathrm{task}\,\mathrm{responses}}{2+\mathrm{joker}\,\mathrm{responses}+\mathrm{task}\,\mathrm{responses}}$$

“task responses” are defined as visits to a corner with at least one nose poke at the task door and “joker responses” as visits with at least one nose poke at the joker door. The ratio R approaches 2 if the responses are primarily at the task side and moves closer to 0 if they are mainly at the joker side. A value of 1 indicates no clear preference for either door. The drinking task ratio was used as a measure of the preference for drinking at task sides, calculated by replacing “responses” with “takes” in the formula above. Accordingly, “task takes” are defined as visits with at least one lick at the task side bottle and “joker takes” as visits with at least one lick at the joker side bottle. In order to avoid bias by the variation of response rates across the light-dark cycle, our calculations for this and other parameters were based on 24 h periods containing equal amounts of light and dark phase. Calculations were done in MS Excel for each individual mouse using an algorithm blind to the group assignment of the evaluated subject.

In addition, the false rate was calculated, representing the percentage of task responses made at incorrect corners, serving as an indicator of learning and overall performance on the task (Ma et al., 2023). Without learning, this rate is typically around 75% ( = chance level). A significant reduction from chance level indicates successful learning of the task rule.

The number of licks per day was monitored continuously and used as indirect measure to detect changes of liquid intake under different experimental conditions. Mice doing less than 100 licks in 24 h were removed from the experiment and placed in standard housing conditions. Body weight was measured regularly during cage cleaning. Main and extension cages were cleaned in alternance to prevent disruption of the familiar environment.

### Statistical analysis

Statistical analyses were performed using R (version 4.4.1, used with packages ggplot2, plyr, mass, reshape2, effectsize, and moments). To evaluate the sex-dependency of dual task motivation, a linear model was used with two between subject factors: group (control group: single motivation protocol = saccharin + water, dual motivation group: dual motivation protocol = saccharin + quinine) and sex (female, male). Neutral and T3 groups were welfare controls used only for specific analyses. For the evaluation of licking, the neutral group (on free adaptation throughout) was added to the group factor. For the evaluation of body weight, the T3 group (in standard type III cages throughout) was added as fourth level to the group factor. Within subject factors were added as needed to explore the dependence of effects on protocol or time. The significance threshold was set at 0.05. Partial ω^2^ served as measure of effect size. Significant interactions were explored by splitting the model. Significant effects of factors with three or more categorical levels were further explored using partial models. *Post hoc* comparisons of group means against chance values were performed using one-sample *t*-tests. The false discovery rate (FDR) control procedure of Benjamini and Hochberg (1995) was applied during *post-hoc* testing. Variables with skewed distributions were subjected to Box-Cox transformation before statistical analysis, as specified in figure legends. Transformation parameters were determined using the R function boxcox. Normality of transformed data was verified visually by plotting model residuals against standard normal quantiles (QQ plot), homoscedascity by plotting residuals against fitted values. All figures show untransformed data. Because the arrangement of groups in cages enforced nesting of both group and sex factors inside cages, scatter plots of individual IntelliCages were compared to verify visually that claimed effects were not contributed by single outlier cages but contributed by both cages representing each factor level combination.

One male mouse of the dual motivation group had to be removed from the experiment during NPA IVa due to insufficient licking. The data of one male mouse of the control group was excluded from analysis of learning tasks because the animal refused to drink saccharin during adaptation. These exclusions were made without considering group memberships of the animals, based on predefined criteria and before the linear models were run on the data.

## Results

### Introduction of quinine during the adaptation phase had a stronger effect on the motivation to respond at task sides in male mice

**FIGURE 3:**
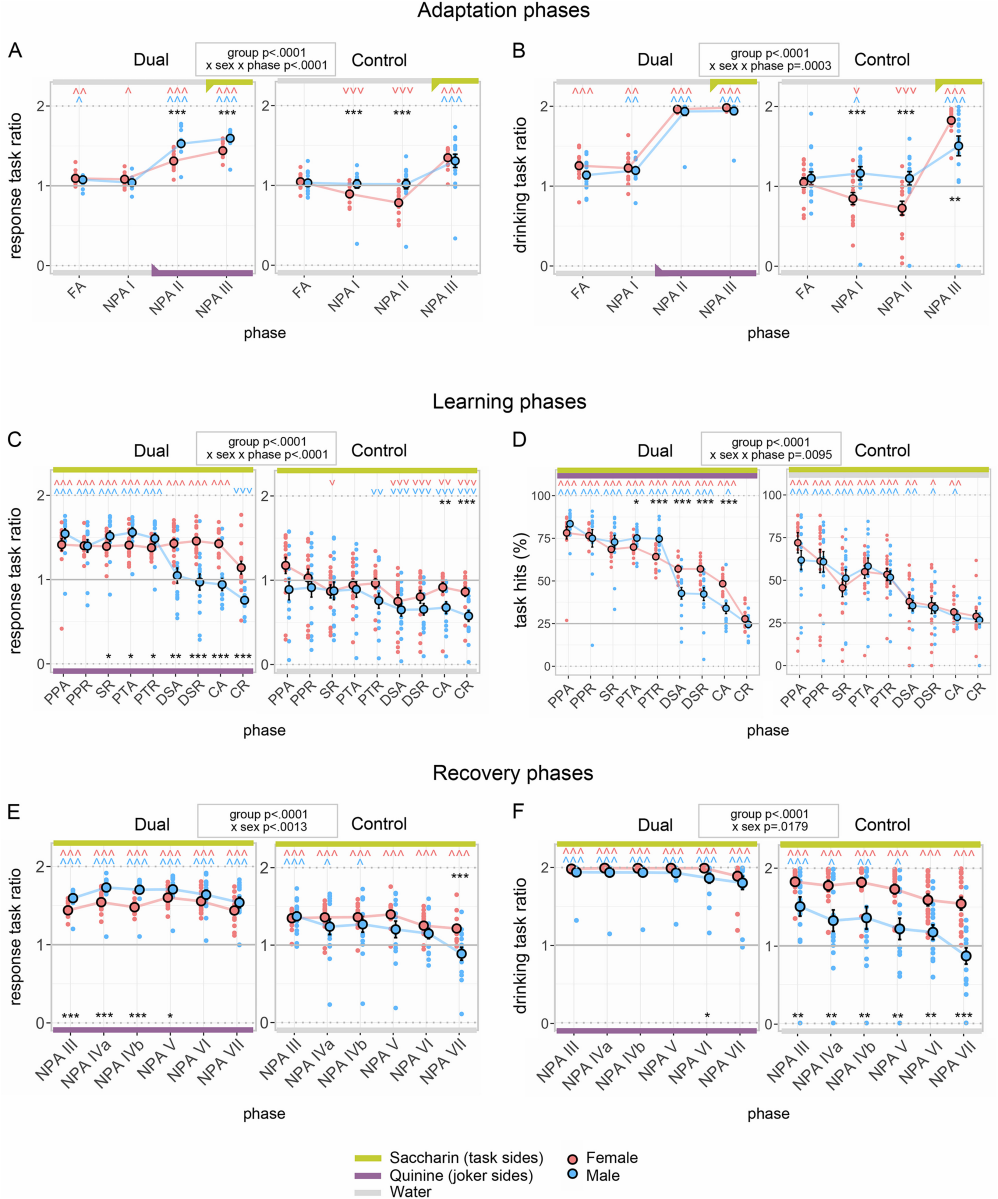
Overview of adaptation, learning and recovery phases (see Figure 2 for protocol details and timeline) showing untransformed data of the last 48 h of each phase for dual (Dual) and single motivation (Control) groups in adjacent panels. For response task ratio **(A,C,E)** and drinking task ratio **(B,F)**, zero indicates total preference for joker door/bottle, 1 means no door/bottle preference, 2 means total preference for task door/bottle. Adaptation phases **(A,B)** consisted of free adaptation (FA), followed by nose poke adaptation I–III (NPA I–III). Learning phases **(C,D)** began with place preference acquisition (PPA), reversal (PPR), and serial reversal (SR), followed by place time acquisition (PTA) and reversal (PTR), diagonal sequencing acquisition (DSA) and reversal (DSR), chaining acquisition (CA) and reversal (CR). Recovery phases **(E,F)** NPA IVa+b, V–VII used the same protocol as NPA III. Asterisks *p < 0.05, **p < 0.01, ***p < 0.001 inside the panel indicate *post hoc* sex differences. Red and blue ∨∧, ∨∨∧∧, ∨∨∨∧∧∧ stand for significant deviation from indifference level 1 **(A–C**, **E,F)** or chance level 25% **(D)** according to one-sample *t*-tests of females and males, respectively. **(A)** The preference to respond to the task sides during adaptation developed after the introduction of the aversive stimulus quinine in NPA II for the dual motivation group and the appetitive stimulus saccharin in NPA III for the control group. The dual motivation group showed a stronger preference to respond to task sides than the control group, and dual motivation males responded more strongly to the task sides than females, as evidenced by a highly significant sex x group x phase interaction as well as a *post hoc* sex effects in the dual motivation group during NPA II and NPA III phases (phase: F3,177 = 175.7, *p* < 0.0001, ω^2^ = 0.64; group: F1,59 = 87.16, *p* < 0.0001, ω^2^ = 0.59 + 0.2000; group × phase: F3,177 = 44.92, *p* < 0.0001, ω^2^ = 0.31; sex: F1,59 = 21.15, *p* < 0.0001, ω^2^ = 0.25 + 0.0977; sex × phase: F3,177 = 12.60, *p* < 0.0001, ω^2^ = 0.10; sex × group: F1,59 = 2.723, ns; sex × group × phase: F3,177 = 7.363, *p* = 0.0001, ω^2^ = 0.02; Box-Cox λ 4.50). **(B)** At the end of the adaptation phases, all groups showed a clear preference to drink at the task sides. This preference was nearly exclusive in the dual motivation group and weaker in the control group, especially in the male controls, who showed the most variable and on average weakest preference for drinking at the task sides (phase: F3,177 = 311.7, *p* < 0.0001, ω^2^ = 0.77; group: F1,59 = 214.5, *p* < 0.0001, ω^2^ = 0.78 + 0.4061; group × phase: F3,177 = 116.1, *p* < 0.0001, ω^2^ = 0.55; sex: F1,59 = 0.2035, ns; sex × phase: F3,177 = 8.508, *p* < 0.0001, ω^2^ = 0.07; sex × group: F1,59 = 1.573, ns; sex × group × phase: F3,177 = 6.690, *p* = 0.0003, ω^2^ = 0.01; Box-Cox λ 4.50). **(C)** The preference to respond at task sides during learning tasks was generally higher in the dual motivation group. Females maintained this preference until the chaining acquisition task, males only until the place time reversal task, responding preferentially at joker sides in the final chaining reversal task. The control group never showed a strong preference for the task sides and instead responded preferentially to the joker sides already during the serial reversal task, with males showing a stronger shift than females (phase: F1,492 = 228.9, *p* < 0.0001, ω^2^ = 0.19 − 0.0519; group: F1,58 = 90.59, *p* < 0.0001, ω^2^ = 0.60 + 0.4883; group × phase: F1,492 = 15.04, *p* = 0.0001, ω^2^ = 0.01; sex: F1,58 = 5.143, *p* = 0.0271, ω^2^ = 0.06-0.1164; sex × phase: F1,492 = 60.05, *p* < 0.0001, ω^2^ = 0.06; sex × group: F1,58 = 0.0000, ns; sex × group × phase: F1,492 = 46.93, *p* < 0.0001, ω^2^ = 0.02; Box-Cox λ 3.00). **(D)** Task hits (responses with at least one nose poke hitting an open task side door) decreased across tasks reflecting their increasing difficulty. Dual motivation improved performance in all tasks except chaining reversal, in the place time task more in males, in subsequent tasks more in females (phase F1,492 = 694.3, *p* < 0.0001, ω^2^ = 0.50 − 5.694; group F1,58 = 40.96, *p* < 0.0001, ω^2^ = 0.40 + 13.60; group × phase F1,492 = 11.19, *p* = 0.0009, ω^2^ = 0.01; sex F1,58 = 0.8551, ns; sex × phase F1,492 = 5.910, *p* = 0.0154, ω^2^ = 0.01; sex × group F1,58 = 0.0721, ns; sex × group × phase F1,492 = 6.776, *p* = 0.0095, ω^2^ < 0.01). **(E)** Preference to respond at task sides during recovery phases was higher in the dual motivation group than in controls. Males of the dual motivation group showed a stronger overall preference than females, while in the control group there was an opposite trend with males unlike females losing saccharin preference across phases (phase: F1,311 = 33.49, *p* < 0.0001, ω^2^ = 0.04 − 0.0263; group: F1,59 = 61.11, *p* < 0.0001, ω^2^ = 0.50 + 0.3574; group × phase: F1,311 = 21.69, *p* < 0.0001, ω^2^ = 0.02; sex: F1,59 = .3294, ns; sex × phase: F1,311 = 12.26, *p* = 0.0005, ω^2^ = 0.01; sex × group: F1,59 = 11.49, *p* = 0.0013, ω^2^ = 0.15; sex × group × phase: F1,311 = 0.8384, ns; Box-Cox λ 2.50). **(F)** Preference to drink at task sides during the recovery phases was higher and more stable in the dual motivation group, with the preference being similar between the sexes. The male controls had a weaker preference than the female controls and lost the preference for drinking at the task sides toward the end of testing (phase: F1,311 = 104.5, *p* < 0.0001, ω^2^ = 0.09 − 0.0547; group: F1,59 = 70.40, *p* < 0.0001, ω^2^ = 0.53 + 0.5225; group × phase: F1,311 = 26.37, *p* < 0.0001, ω^2^ = 0.02; sex: F1,59 = 15.79, *p* = 0.0002, ω^2^ = 0.20 − 0.2494; sex × phase: F1,311 = 3.550, *p* = 0.0605, ω^2^ = 0.00; sex × group: F1,59 = 5.936, *p* = 0.0179, ω^2^ = 0.07; sex × group × phase: F1,311 = 0.0335, ns; Box-Cox λ 4.50).

During the initial 6 days in the IntelliCage, the mice were in free adaptation (FA) to habituate to the new environment. All eight doors in the four IntelliCage corners remained open and provided unrestricted access to plain water (Figure 2). The mice habituated to the new environment as expected, as response frequency (visits with at least one nose poke) decreased robustly across days, with no evidence for a group effect (data not shown). Although the task and joker sides were identical in terms of door mechanism and stimuli, the mice developed slight spontaneous preferences to respond at prospective task or joker sides (Figure 3A).

The FA phase was followed by a 10-day nose poke adaptation (NPA) protocol, divided into three phases (NPA I–III, Figure 2), to train the mice on the door mechanism for accessing bottles and to habituate them to the taste stimuli. Each time a mouse entered a corner, the door on the joker side automatically opened for 3 s, giving free access to the joker side bottle. To access the task side bottle, the mice had to actively perform a nose poke to open the task side door. Quinine and saccharin solutions were stepwise introduced in NPA II and III, respectively.

In response to the introduction of quinine, the dual motivation group established a clear preference to respond to task sides, with the effect being stronger in males than females (Figure 3A). As reaction to the introduction of saccharin, the dual motivation group further intensified responding to task sides and also the control group developed clear preference to respond to task sides. This preference remained stronger and more consistent in the dual motivation group and strongest in dual motivation males (Figure 3A). Drinking preferences overall developed in parallel with responding preferences (Figure 3B) but tended to be more extreme, reflecting ongoing lickless exploratory responding on non-preferred sides. By the end of the adaptation, all groups had established a clear preference for drinking at task sides (Figure 3B).

Taken together, these results demonstrate that in absence of a task challenge the disincentive stimulus quinine alone or in combination with saccharin had a stronger effect on the motivation for task side responses in males than in females. The motivational effect of saccharin alone on responding in the control group was similar in both sexes.

### The dual motivation protocol improved motivation and learning in all but the last task in females but became ineffective during the diagonal sequencing and chaining tasks in males

**FIGURE 4:**
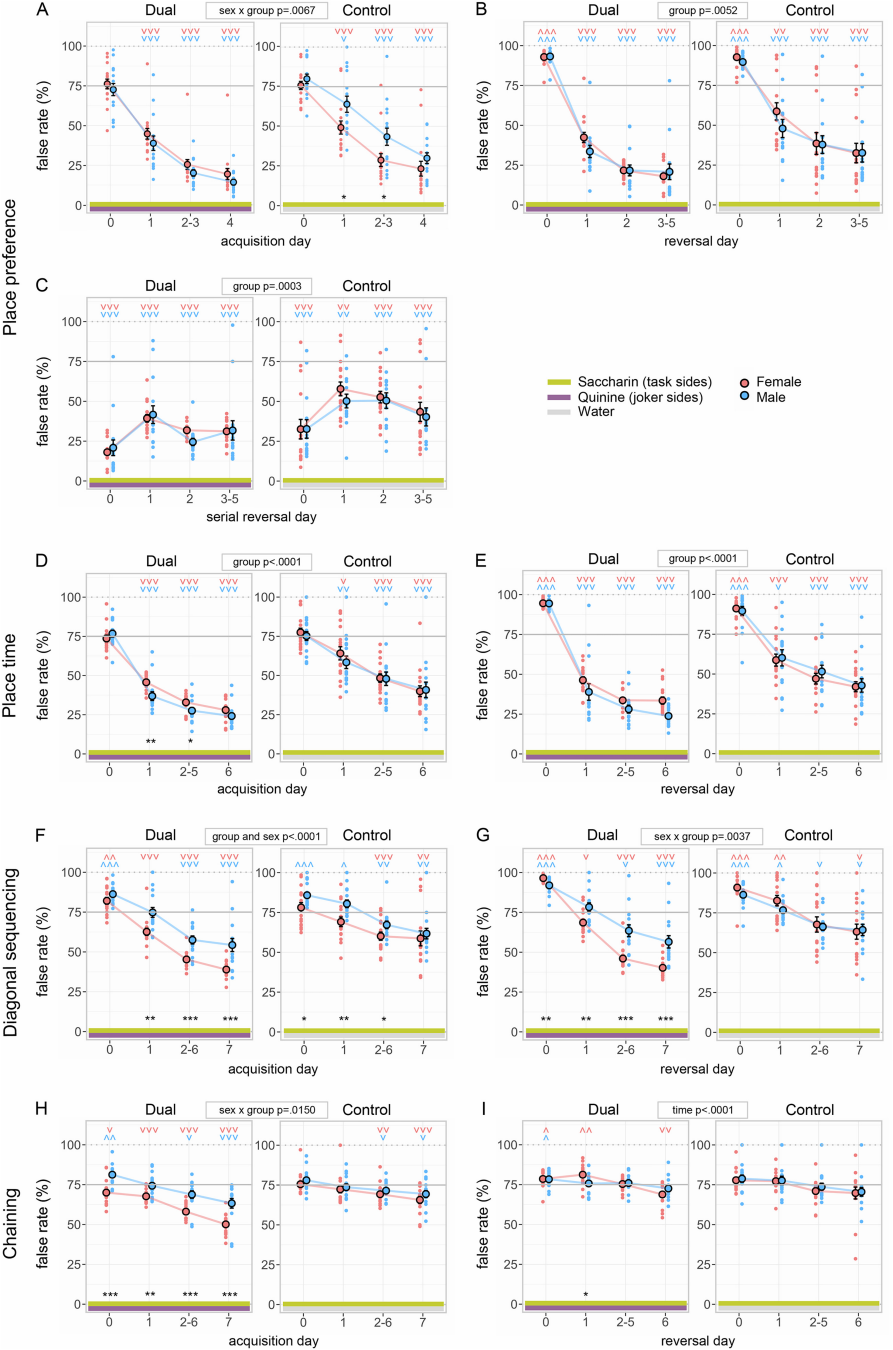
Task performance across learning tasks for dual (Dual) and single motivation (Control) groups shown in adjacent panels using untransformed data. Black asterisks *p < 0.05, **p < 0.01, ***p < 0.001 inside the panel indicate *post hoc* sex differences. Red and blue ∨∧, ∨∨∧∧, ∨∨∨∧∧∧ stand for significant deviation from chance level 75% according to one-sample *t*-tests of females and males, respectively. Day 0 indicates last day of previous NPA for acquisition **(A,D,F,H)** and last day of the previous task phase for reversal **(B,C,E,G,I)**. **(A)** In place preference acquisition, the error rates decreased rapidly in both groups. Male mice of the control group showed poorer learning and performance compared to males in the dual motivation group and females overall (time: F3,177 = 499.2, *p* < 0.0001, ω^2^ = 0.70; group: F1,59 = 13.22, *p* = 0.0006, ω^2^ = 0.17 − 11.18; group × time: F3,177 = 7.085, *p* = 0.0002, ω^2^ = 0.03; sex: F1,59 = 0.2587, ns; sex × time: F3,177 = 0.3966, ns; sex × group: F1,59 = 7.907, *p* = 0.0067, ω^2^ = 0.10; sex × group x time: F3,177 = 3.458, *p* = 0.0176, ω^2^ = 0.00; Box-Cox λ 0.50). **(B)** During reversal, the error rates decreased rapidly in both groups but with better learning and performance in the dual motivation group and without an evidence of a sex effect (time: F3,177 = 263.3, *p* < 0.0001, ω^2^ = 0.66; group: F1,59 = 8.439, *p* = 0.0052, ω^2^ = 0.11 − 10.26; group × time: F3,177 = 4.592, *p* = 0.0040, ω^2^ = 0.03; sex: F1,59 = 0.2619, ns; sex × time: F3,177 = 1.838, ns; sex × group: F1,59 = 0.2304, ns; sex × group × time: F3,177 = 0.2873, ns; Box-Cox λ 0.50). **(C)** During serial reversal, error rates decreased in both groups despite the daily change of the target, indicating learning of a general task rule. The dual motivation group showed superior learning and performance. No sex effects were observed (time: F3,177 = 24.84, *p* < 0.0001, ω^2^ = 0.15; group: F1,59 = 15.02, *p* = 0.0003, ω^2^ = 0.19 − 13.81; group × time: F3,177 = 3.557, *p* = 0.0155, ω^2^ = 0.02; sex: F1,59 = 0.0002, ns; sex × time: F3,177 = 0.2977, ns; sex × group: F1,59 = 0.6101, ns; sex × group × time: F3,177 = 0.8810, ns). **(D)** In place time acquisition, both groups learnt well. The dual motivation group showed better learning and performance than controls, in particular males (time: F3,174 = 320.3, *p* < 0.0001, ω^2^ = 0.66; group: F1,58 = 36.55, *p* < 0.0001, ω^2^ = 0.37 − 14.52; group × time: F3,174 = 18.63, *p* < 0.0001, ω^2^ = 0.10; sex: F1,58 = 2.738, ns; sex × time: F3,174 = 2.719, *p* = 0.0461, ω^2^ = 0.01; sex × group: F1,58 = 0.6241, ns; sex × group × time: F3,174 = 1.257, ns; Box-Cox λ 0.50). **(E)** During reversal, error rates decreased across task days. The dual motivation group showed clearly better learning and performance compared to the control group (time: F3,174 = 467.5, *p* < 0.0001, ω^2^ = 0.78; group: F1,58 = 24.24, *p* < 0.0001, ω^2^ = 0.28 − 11.11; group × time: F3,174 = 18.69, *p* < 0.0001, ω^2^ = 0.12; sex: F1,58 = 0.9540, ns; sex × time: F3,174 = 0.5982, ns; sex × group: F1,58 = 2.410, ns; sex × group × time: F3,174 = 1.399, ns). **(F)** In diagonal sequencing acquisition, the dual motivation group outperformed the control group and females outperformed males (time: F3,174 = 172.9, *p* < 0.0001, ω^2^ = 0.57; group: F1,58 = 18.36, *p* < 0.0001, ω^2^ = 0.22 − 8.356; group × time: F3,174 = 16.70, *p* < 0.0001, ω^2^ = 0.11; sex: F1,58 = 23.70, *p* < 0.0001, ω^2^ = 0.27 + 9.498; sex × time: F3,174 = 2.388, *p* = 0.0706, ω^2^ = 0.01; sex × group: F1,58 = 1.546, ns; sex × group × time: F3,174 = 3.554, *p* = 0.0156, ω^2^ = 0.01; Box-Cox λ 0.50). **(G)** During reversal, females of the dual motivation group outperformed all other groups (time: F3,174 = 212.4, *p* < 0.0001, ω^2^ = 0.60; group: F1,58 = 14.85, *p* = 0.0003, ω^2^ = 0.19 − 8.395; group x time: F3,174 = 21.94, *p* < 0.0001, ω^2^ = 0.13; sex: F1,58 = 5.913, *p* = 0.0181, ω^2^ = 0.08 + 5.300; sex × time: F3,174 = 11.51, *p* < 0.0001, ω^2^ = 0.07; sex × group: F1,58 = 9.143, *p* = 0.0037, ω^2^ = 0.12; sex × group × time: F3,174 = 4.867, *p* = 0.0028, ω^2^ = 0.01; Box-Cox λ 0.50). **(H)** In chaining acquisition, females of the dual motivation group showed better learning and performance than female controls. Performance of males was overall worse and showed no evidence for a group effect (time: F3,174 = 79.83, *p* < 0.0001, ω^2^ = 0.32; group: F1,58 = 9.513, *p* = 0.0031, ω^2^ = 0.12 − 4.896; group × time: F3,174 = 7.897, *p* < 0.0001, ω^2^ = 0.04; sex: F1,58 = 16.92, *p* = 0.0001, ω^2^ = 0.21 + 6.534; sex × time: F3,174 = 1.376, ns; sex × group: F1,58 = 6.282, *p* = 0.0150, ω^2^ = .008; sex × group × time: F3,174 = 0.2985, ns; Box-Cox λ 1.50). **(I)** During reversal, error rates decreased modestly across task days and fell slightly below chance until the end of the task. There was no evidence for group or sex effects (time: F3,174 = 23.76, *p* < 0.0001, ω^2^ = 0.13; group: F1,58 = 0.3777, ns; group × time: F3,174 = 1.029, ns; sex F1,58 = 0.0480, ns; sex × time: F3,174 = 1.801, ns; sex × group: F1,58 = 0.2180, ns; sex × group × time: F3,174 = 1.958, ns; Box-Cox λ 1.50).

**FIGURE 5:**
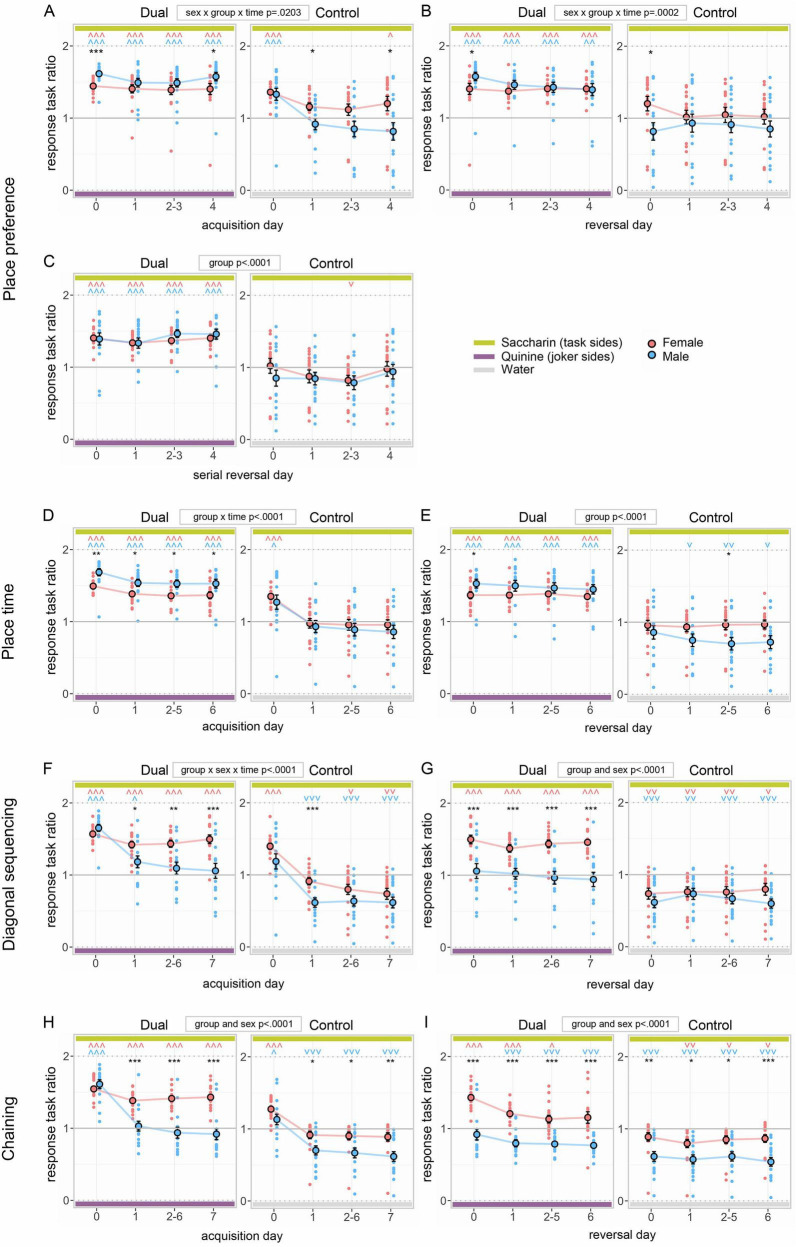
Task motivation across learning tasks for dual (Dual) and single motivation (Control) groups shown in adjacent panels using untransformed data. Zero indicates total preference for joker door/bottle, 1 means no door/bottle preference, 2 means total preference for task door/bottle. Black asterisks *p < 0.05, **p < 0.01, ***p < 0.001 inside the panel indicate *post hoc* sex differences. Red and blue ∨∧, ∨∨∧∧, ∨∨∨∧∧∧ stand for significant deviation from chance level 1 according to one-sample t-tests of females and males, respectively. Day 0 indicates last day of previous NPA for acquisition **(A,D,F,H)** and last day of the previous task phase for reversal **(B,C,E,G,I)**. **(A)** In place preference acquisition, the dual motivation group showed a persistent preference to respond to the task sides, which was more pronounced in males. Controls strongly reduced responding at task sides with task begin, with males losing it completely (time: F3,177 = 30.76, *p* < 0.0001, ω^2^ = 0.11; group F1,59 = 46.62, *p* < 0.0001, ω^2^ = 0.43 + 0.3924; group × time: F3,177 = 10.49, *p* < 0.0001, ω^2^ = 0.04; sex F1,59 = 0.2583, ns; sex × time: F3,177 = 6.105, *p* = 0.0006, ω^2^ = 0.02; sex × group: F1,59 = 8.311,*p* = 0.0055, ω^2^ 0.11; sex × group × time: F3,177 = 3.350, *p* = 0.0203, ω^2^ = 0.01; Box-Cox λ 3.50). **(B)** During reversal, the dual motivation group unlike controls maintained a persistent preference for the task doors (time: F3,177 = 8.099, *p* < 0.0001, ω^2^ = 0.02; group: F1,59 = 36.03, *p* < 0.0001, ω^2^ = 0.36 + 0.4674; group × time: F3,177 = 0.9894, ns; sex: F1,59 = 0.0034, ns; sex × time: F3,177 = 1.939, ns; sex × group: F1,59 = 2.946, *p* = 0.0913, ω^2^ = 0.03; sex × group × time: F3,177 = 7.015, *p* = 0.0002, ω^2^ = 0.01; Box-Cox λ 3.50). **(C)** During serial reversal, the dual motivation group maintained its preference to respond at task sides, while controls transiently responded preferentially at joker sides (time: F3,177 = 8.729, *p* < 0.0001, ω^2^ = 0.02; group: F1,59 = 57.04, *p* < 0.0001, ω^2^ = 0.48 + 0.5157; group × time: F3,177 = 3.735, *p* = 0.0123, ω^2^ = 0.01; sex: F1,59 = 0.1456, ns; sex × time: F3,177 = 2.145, *p* = 0.0963, ω^2^ = 0.00; sex × group: F1,59 = 0.6689, ns; sex × group × time: F3,177 = 0.4386, ns; Box-Cox λ 3.00). **(D)** In place time acquisition, the dual motivation group persistently preferred to respond at task sides, males more strongly than females, while controls lost this preference at task begin (time: F3,174 = 81.92, *p* < 0.0001, ω^2^ = 0.21; group: F1,58 = 71.22, *p* < 0.0001, ω^2^ = 0.54 + 0.4613; group × time: F3,174 = 10.54, *p* < 0.0001, ω^2^ = 0.03; sex: F1,58 = 3.266, *p* = 0.0759, ω^2^ = 0.04 + 0.0988; sex × time: F3,174 = 0.3385, ns; sex × group: F1,58 = 5.049, *p* = 0.0285, ω^2^ = 0.06; sex × group × time: F3,174 = 0.3406, ns; Box-Cox λ 2.50). **(E)** During reversal, the dual motivation group maintained a stable preference for task sides as during acquisition, while controls lacked such a preference, with males even switching to preferential responding at joker sides (time: F3,174 = 2.880, *p* = 0.0375, ω^2^ = 0.00; group: F1,58 = 78.10, *p* < 0.0001, ω^2^ = 0.56 + 0.5529; group × time: F3,174 = 0.7984, ns; sex: F1,58 = 0.1738, ns; sex × time: F3,174 = 3.566, *p* = 0.0154, ω^2^ = 0.01; sex × group: F1,58 = 5.322, *p* = 0.0246, ω^2^ = 0.07; sex × group × time: F3,174 = 0.5416, ns). **(F)** In diagonal sequencing acquisition, only females of the dual motivation group maintained a stable preference for responding at task sides, with males of the group rapidly reaching indifference. Controls switched to preferential responding a joker sides, males more rapidly than females (time: F3,174 = 128.4, *p* < 0.0001, ω^2^ = 0.36; group: F1,58 = 68.47, *p* < 0.0001, ω^2^ = 0.53 + 0.4928; group × time: F3,174 = 10.64, *p* < 0.0001, ω^2^ = 0.04; sex: F1,58 = 11.88, *p* = 0.0011, ω^2^ = 0.15 − 0.2054; sex × time: F3,174 = 6.893, *p* = 0.0002, ω^2^ = 0.03; sex × group: F1,58 = 0.2343, ns; sex × group × time: F3,174 = 10.60, *p* < 0.0001, ω^2^ = 0.02). **(G)** During reversal, only females of the dual motivation group responded at task sides with consistent preference. Their male peers responded without evident preference throughout the phase. Controls preferentially responded at joker sides (time: F3,174 = 0.4756, ns; group: F1,58 = 58.94, *p* < 0.0001, ω^2^ = 0.49 + 0.5088; group × time: F3,174 = 2.778, *p* = 0.0427, ω^2^ = 0.01; sex: F1,58 = 16.61, *p* = 0.0001, ω^2^ = 0.21 − 0.2703; sex × time: F3,174 = 3.287, *p* = 0.0221, ω^2^ = 0.01; sex × group: F1,58 = 7.003, *p* = .0105, ω^2^ = 0.09; sex × group × time: F3,174 = 0.1303, ns). **(H)** In chaining acquisition, only females of the dual motivation group maintained a stable preference for responding at task sides. Males of the dual motivation group like female controls instantly switched to indifferent responding. Male controls were the only group showing a strong preference to respond at joker sides from the onset of the task (time: F3,174 = 196.4, *p* < 0.0001, ω^2^ = 0.39; group: F1,58 = 57.03, *p* < 0.0001, ω^2^ = 0.48 + 0.3892; group × time: F3,174 = 0.7851, ns; sex: F1,58 = 24.94, *p* < 0.0001, ω^2^ = 0.29 − 0.2575; sex × time: F3,174 = 31.24, *p* < 0.0001, ω^2^ = 0.09; sex × group: F1,58 = 1.445, ns; sex × group × time: F3,174 = 12.07, *p* < 0.0001, ω^2^ = 0.01). **(I)** During reversal, females of the dual motivation group began to lose their clear preference to respond at task sides and approached indifference. All other groups switched to preferential responding at task sides, males more strongly than females (time: F3,174 = 19.55, *p* < 0.0001, ω^2^ = 0.05; group: F1,58 = 34.36, *p* < 0.0001, ω^2^ = 0.36 + 0.3026; group × time: F3,174 = 9.727, *p* < 0.0001, ω^2^ = 0.03; sex: F1,58 = 41.63, *p* < 0.0001, ω^2^ = 0.40 − 0.3333; sex × time: F3,174 = 2.609, *p* = 0.0532, ω^2^ = 0.00; sex × group: F1,58 = 2.513, ns; sex × group × time: F3,174 = 1.953, ns).

Following adaptation phases, learning tasks began (Figure 2). In the place preference acquisition (PPA) task, drinking was permitted in all corners at joker sides, while saccharin at task sides was only accessible in a single permanently correct corner. Despite the simplicity of the task, control males demonstrated poorer learning and performance than control females (Figure 4A). Only the dual motivation group maintained a robust motivation to respond at task sides (Figures 3C, 5A), with the loss of motivation being more pronounced in males of the control group than in females. In the subsequent place preference reversal (PPR) task, saccharin at task sides was accessible in a corner opposite the acquisition target. The dual motivation group again outperformed controls but no sex differences were observed (Figure 4B). And again only the dual motivation group maintained a robust motivation to respond at task sides (Figures 3C, 5B). The place preference task ended with a serial reversal (SR) phase in which the target changed every 24 h according to a pseudorandom pattern. As during previous phases, the dual motivation group outperformed controls (Figure 4C). Only the dual motivation group maintained a robust preference for the task sides, whereas the control animals tended to favor the joker sides without evidence for sex differences (Figures 3C, 5C).

The place time acquisition (PTA) task involved saccharin targets alternating at 2 a.m. and 2 p.m. between two adjacent corners. The dual motivation group clearly outperformed controls (Figure 4D), with males of the dual motivation group learning slightly faster than females of the group. As in in the previous task, only the dual motivation group maintained a robust preference to respond at task sides (Figures 3C, 5D), with males profiting more than females from the dual motivation protocol. In the place time reversal (PTR) task, saccharin targets alternated at 2 a.m. and 2 p.m. between corners opposite those in the acquisition phase. The dual motivation group outperformed controls regardless of sex (Figure 4E). As during acquisition, only the dual motivation group maintained a robust preference to respond at task sides (Figures 3C, 5E), with males still profiting more than females from the dual motivation protocol and control males switching to preferential responding at joker sides.

In the diagonal sequencing acquisition (DSA) task, the correct corner switched diagonally after each correct task response. The dual motivation group demonstrated better learning and performance than controls and females performed better than males (Figure 4F). Only females of the dual motivation group maintained preferential responding at task sides with males approaching indifference. In contrast to previous tasks, males now profited less from the dual motivation protocol. Controls switched to preferential responding at joker sides, males somewhat more rapidly than females (Figures 3C, 5F). During the diagonal sequencing reversal (DSR) task, the two previously incorrect corners became correct, again switching diagonally after each correct task response. The dual motivation group learned better than the control group (Figure 4G), but the dual motivation effect was smaller in males than in females. Group and sex effects on task motivation were similar to the acquisition phase (Figures 3C, 5G).

In the chaining acquisition (CA) task, the correct corner shifted after each task response in a clockwise or counterclockwise sequence. Females of the dual motivation group outperformed all other subgroups with no evidence for males profiting from the dual motivation protocol (Figure 4H). As in the previous task, females of the dual motivation group were the only subgroup to maintain preferential responding at task sides (Figures 3C, 5H). The other subgroups responded indifferently, control males even preferentially at joker sides. The chaining reversal (CR) task reversed the shift direction of the target. In this most difficult task, there were no significant group or sex differences on performance (Figure 4I). Only females of the dual motivation group showed some initial preference to respond to task sides, while the other subgroups favored responding to joker sides, males more strongly than females (Figures 3C, 5I).

To summarize, both dual motivation and control groups showed learning and task rule acquisition up to the CA task, with the dual motivation group consistently outperforming controls (Figure 3D). In the place preference and place time tasks, males of the dual motivation group performed at par with or even slightly better than female peers. This changed with the diagonal sequencing task (Figure 3D). From this task onward, females of the dual motivation group outperformed their male peers, except in the last and most difficult chaining reversal task where learning was poor overall and no effect of the dual motivation protocol was evident anymore. This was paralleled by task and sex dependent effects of the dual motivation protocol on the preference to respond for saccharin at task sides. In several tasks, male controls were more prone to lose preferential responding at task sides compared to females in the group. In the place preference and place time tasks, this was compensated or even overcompensated in the dual motivation group, but this compensation effect broke down with the onset of the diagonal sequencing task, with males of the dual motivation group no longer preferentially responding at task sides.

### Quinine stabilized and equalized the motivation of male and female mice to respond at task sides during recovery phases

To prevent interference between learning rules, a recovery phase with an NPA protocol was introduced after each learning task (see Figure 2 for an overview of the timeline). During the recovery phases, the preference to respond to the task sides was stronger and more sustained in the dual motivation group compared to controls, with this effect being more pronounced in males than in females (Figure 3E). In males of the control group, the task side preference was particularly variable and decreased more strongly across phases, reaching indifference by the end of testing. One control male very early developed an extreme preference for responding to joker sides. The preference to drink at task sides remained nearly exclusive throughout the observation period in most animals of the dual motivation group (Figure 3F). In controls, this preference was more variable and decreased across phases, more strongly in males which unlike females reached the point of indifference by the end of testing.

### There was no evidence for a restriction of drinking or weight gain by the exposure to taste stimuli and learning challenges

**FIGURE 6:**
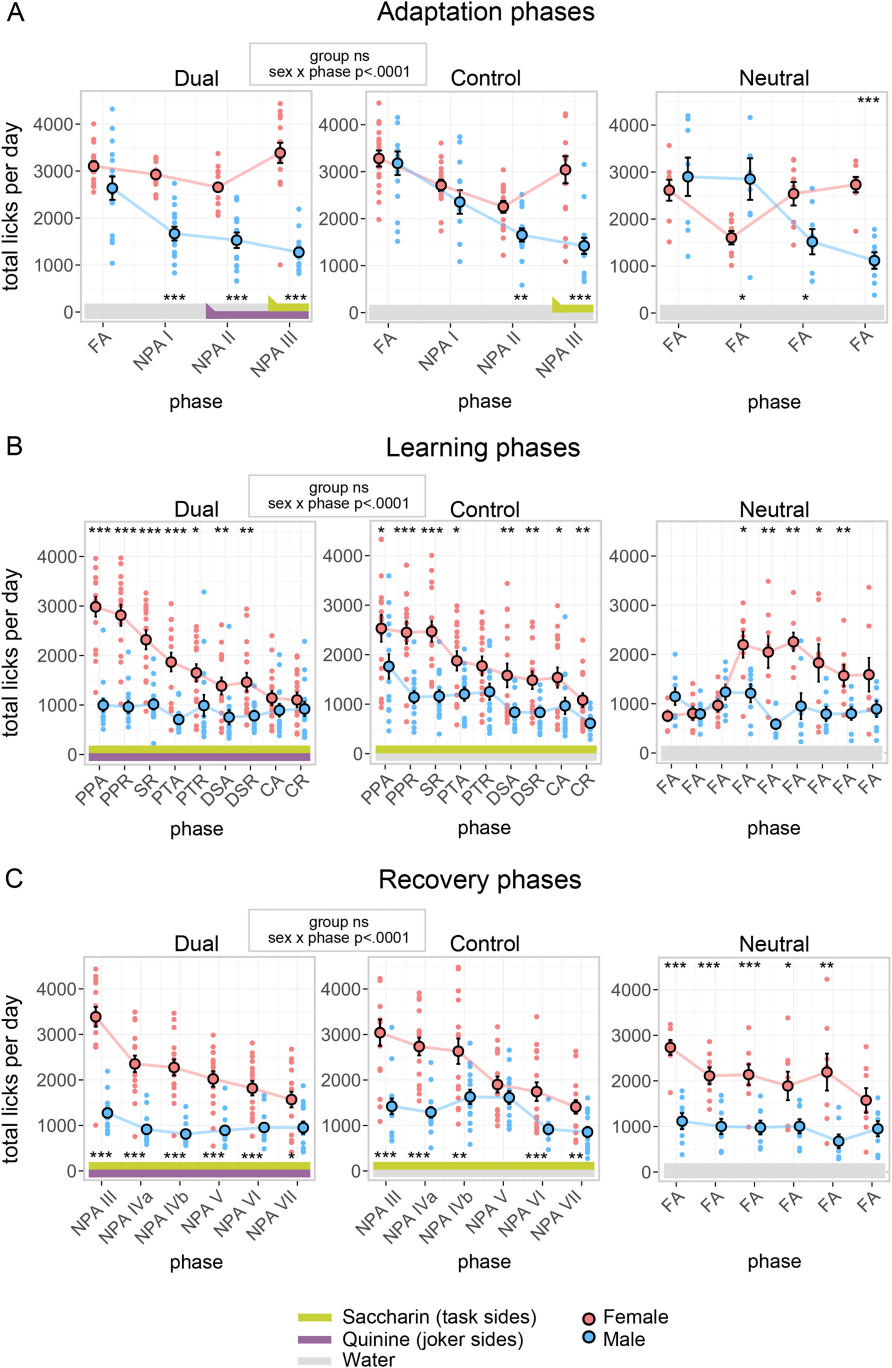
Overview of adaptation, learning and recovery phases showing untransformed lick numbers of the last 48 h of each phase for dual (Dual) and single motivation (Control) groups in adjacent panels, along with data of a group (Neutral) which remained on the free adaptation protocol during the entire study. Adaptation phases **(A)** consisted of free adaptation (FA), followed by nose poke adaptation I-III (NPA I–III). Learning phases **(B)** began with place preference acquisition (PPA), reversal (PPR), and serial reversal (SR), followed by place time acquisition (PTA) and reversal (PTR), diagonal sequencing acquisition (DSA) and reversal (DSR), chaining acquisition (CA) and reversal (CR). Recovery phases **(C)** NPA IVa+b, V–VII used the same protocol as NPA III. Black asterisks *p < 0.05, **p < 0.01, ***p < 0.001 inside the panel indicate *post hoc* sex differences. **(A)** Across adaptation phases, lick numbers decreased continuously in males but only transiently in females, with the pattern being more pronounced in the dual motivation group (phase: F3,177 = 38.47, *p* < 0.0001, ω^2^ = 0.24; group: F1,59 = 0.2381, ns; group × phase: F3,177 = 3.273, *p* = 0.0225, ω^2^ = 0.02; sex: F1,59 = 59.09, *p* < 0.0001, ω^2^ = 0.49 − 958.3; sex × phase: F3,177 = 21.69, *p* < 0.0001, ω^2^ = 0.15; sex × group: F1,59 = 5.012, *p* = 0.0290, ω^2^ = 0.06; sex × group × phase: F3,177 = 0.4369, ns). The neutral group showed overall similar lick numbers and change across phases, except for nose poke adaptation I, when females licked less than males (group F2,73 = 0.8385, ns; group × time bin F2,152 = 1.508, ns; sex × group F2,73 = 5.737 *p* = 0.0049 ω^2^ = 0.11; sex × group × time bin F2,152 = 8.459 *p* = 0.0003 ω^2^ = 0.02). **(B)** During learning phases, females initially showed much higher lick numbers than males, but this gap narrowed over time due to a stronger decrease in lick numbers in females (phase: F1,492 = 313.8, *p* < 0.0001, ω^2^ = 0.21-135.2; group: F1,58 = 0.7717, ns; group × phase: F1,492 = 1.069, ns; sex: F1,58 = 43.00, *p* < 0.0001, ω^2^ = 0.41 − 884.4; sex × phase: F1,492 = 70.24, *p* < 0.0001, ω^2^ = 0.06; sex × group: F1,58 = .6311, ns; sex × group × phase: F1,492 = 25.90, *p* < 0.0001, ω^2^ = 0.01). The neutral group showed a similar pattern, except for the first three phases when females and males showed similar lick numbers (group F2,72 = 1.230, ns; group × time bin F2,618 = 49.56, *p* < 0.0001; ω^2^ = 0.07; sex × group F2,72 = 0.6613, ns; sex × group × time bin F2,618 = 52.27, *p* < 0.0001, ω^2^ = 0.04; Box-Cox λ 0.50). **(C)** During recovery phases, females initially showed higher lick numbers than males, but this gap narrowed over time due to a stronger decrease in lick numbers in females (phase: F1,311 = 210.8, *p* < 0.0001, ω^2^ = 0.21-202.8; group: F1,59 = 1.369, ns; group × phase: F1,311 = 3.879, *p* = 0.0498, ω^2^ = 0.00; sex: F1,59 = 74.50, *p* < 0.0001, ω^2^ = 0.55 − 1144; sex × phase: F1,311 = 37.13, *p* < 0.0001, ω^2^ = 0.04; sex × group: F1,59 = 1.972, ns; sex × group × phase: F1,311 = 1.440, ns). Similar licking behaviors were observed in the neutral group (group F2,73 = 1.458, ns; group × time bin F2,389 = 4.318, *p* = 0.0140, ω^2^ = 0.01; sex × group F2,73 = 1.080, ns; sex × group × time bin F2,389 = 1.711 ns; Box-Cox λ 0.50).

During the initial adaptation, the number of licks decreased in both groups, independently of the introduction of quinine in the dual motivation group during NPA II. Males showed a greater decline and did not recover when saccharin was added in both groups in NPA III (Figure 6A). Across the learning and recovery phases, the number of licks decreased further. Because this decrease was stronger in females than in males, their lick numbers became more similar with time (Figures 6B, C). No consistent group effects were observed during the learning or recovery phases. The neutral group which served as welfare control and was not exposed to taste stimuli or learning challenges had lick numbers in the same range as the dual motivation and control group, with mostly similar sex differences and time effects. These observations suggest that neither learning challenges nor exposure to taste stimuli, in particular quinine, caused a relevant restriction in drinking.

The body weight of the mice in all groups increased steadily in the expected range with time, indicating that the IntelliCage protocols had no negative effects on the wellbeing of the mice. There was no evidence for a weight difference between the dual motivation and control group. Male and female mice in the T3 group which served as welfare control gained slightly more weight, which was probably due to lower physical activity under less enriched housing conditions and a smaller living space (Supplementary Figure 1).

## Discussion

In an earlier study (Nigri et al., 2024) we had observed that male mice were less motivated than females to learn spatial IntelliCage tasks in which water restriction was prevented by making plain water freely available and offering mice saccharin sweetened water as sole learning incentive and rewards for responses in correct corners. Here, we investigated whether dual motivation protocols in which we made the permanently available water less attractive by adding quinine as a second disincentive stimulus (Ma et al., 2023) would suffice to eliminate this sex difference without reintroducing a component of water restriction to the task. We found that dual motivation protocols produced the desired effect, provided that location of spatial learning targets remained stable over time.

We systematically compared motivation to engage in the task as well as learning performance of male and female C57BL/6J mice in a range of spatial tasks in IntelliCage using either dual (saccharin versus quinine) or single (controls, saccharin versus plain water) motivation protocols. Confirming past observations (Nigri et al., 2024), males and females of the control group were similarly motivated to respond for saccharin (Pelz et al., 1973; Tordoff, 2007; Treesukosol et al., 2009) before the onset of learning challenges. However, males were less able than females to maintain this motivation under the challenge of increasingly difficult learning tasks. As in the previous study, this was associated with a trend toward poorer performance in some of the tasks. In line with our first exploration of dual motivation protocols for female mice in the IntelliCage (Ma et al., 2023), rendering the permanently available water less attractive by adding quinine as a second, disincentive stimulus strongly improved task engagement and performance of female mice throughout the task battery, except in the most difficult task chaining reversal. As intended, this occurred without obviously compromising fluid intake or weight gain.

As judged from drinking choices during adaptation phases and confirming reports in the literature (Thiele et al., 2002; Fulenwider et al., 2019), quinine was similarly aversive for males and females. In our study, it had an even stronger boosting effect on the rate of responding for saccharin in males than in females, not only during adaptation, but also later during learning tasks and recovery phases between tasks. As a consequence, males of the dual motivation group no longer performed more poorly in learning tasks and even outperformed females of the group in some of them. Thus, introducing dual motivation was sufficient to eliminate the motivational disadvantage present in males of the control group. As in females, there was no evidence for a negative effect of the added disincentive stimulus on lick counts as indirect estimate of fluid intake or weight gain of male mice.

However, this strong boosting effect of the disincentive quinine stimulus on task motivation and performance of males collapsed at the transition from the place time to the diagonal sequencing task without recovering later on. This strikingly sharp transition contrasted with the gradual degradation of motivation and performance in females and male controls which paralleled gradually increasing task difficulty, and prompted the question what else than general task difficulty could be responsible for the sudden collapse of motivation and performance in the male dual motivation group at this specific point. Comparison of the task rules reveals that the protocols running before the performance collapse of the male dual motivation group (place preference and place time task) have in common that the rewarded target remains stable for 12 h to several days and is not affected by the responses of the mice. Thus, these tasks impose a win-stay strategy and mainly depend on reference memory. By contrast, learning tasks after the collapse point (diagonal sequencing and chaining task) are characterized by rewarded targets that shift frequently and in response to the choices of the animals. They impose a flexible win-shift strategy and challenge working memory because whether a corner is correct or not depends on recent choices.

Reports of male or female superiority in conventional behavioral tests of spatial cognition are contradictory and remain controversial (Jonasson, 2005; Fritz et al., 2017; Melgar-Locatelli et al., 2024) with a potential small advantage of male mice in some studies of spatial working memory, for example in the 8-arm radial (Jonasson, 2005) or Y-maze (Lou et al., 2025). Published (Weyer et al., 2011; Steubler et al., 2021; Brilkova et al., 2022; Erdinger et al., 2022) and unpublished studies of our laboratory using the 8-arm radial and T-maze tests of spatial working memory never revealed consistent sex differences. If water restriction is used in IntelliCage to enforce learning, male mice perform well (Endo et al., 2011) or even slightly better than females (Aung et al., 2016) in diagonal sequencing as well as chaining (Fischer et al., 2017) tasks. These observations argue against weaker working-memory being responsible for the sharp performance breakdown of male mice in the dual motivation group and point to motivational factors as a more likely explanation.

The importance of sex differences in motivation and value-based decision making is increasingly recognized (Youn et al., 2012; Orsini and Setlow, 2017; Shansky, 2018; Grissom and Reyes, 2019; Chen et al., 2021a,b) and sex differences at cellular level have been identified in circuits that control decision-making in mice (Cox et al., 2023). A sex-specific valuation of incentive and disincentive stimuli during learning may at least in part have accounted for the relatively constant or gradually changing sex differences in the control group. However, it is unlikely to account for the sharp breakdown of motivation and performance of male mice in the dual motivation group, because this breakdown occurred at a time point at which there was neither a change of stimulus configuration nor an abrupt increase of general task difficulty. Rather, the motivational factors explaining the sudden breakdown may be more specifically related to the transition from stable to frequently changing target corners that occurred precisely at this point.

It is well-documented that scent signals play an important role in intermale competition (Hurst and Beynon, 2004). Of particular interest for spatial learning tasks in the social context of IntelliCage are reports of social scent-induced place preference (Roberts et al., 2012) and identification of a circuit potentially mediating pheromonal shaping of hippocampal learning (Villafranca-Faus et al., 2021). These reports led us to speculate that the flexible win-shift strategy imposed by the learning rules of IntelliCage tasks after the performance breakdown may have competed with spatial preferences of male mice that were motivated by social hierarchy or territorial behavior. This could also account for the observation that the motivation of males in the dual motivation group was instantly restored when task rules were released in the recovery phases. By contrast, the spatial stability of targets during earlier learning tasks may have given the group sufficient time to adapt its socio-spatial organization to the configuration of targets. This is only a hypothesis and we cannot fully exclude alternative explanations based on the results of this study. Whether our favored hypothesis is true remains to be established empirically, for example by running our dual motivation protocols in parallel with socially and singly housed male mice. Follow-up experiments could also establish whether rendering the permanently available water even less attractive, for example by combining quinine with a higher number of nose pokes needed to gain access or with a gambling-like mechanism (Ma et al., 2023) would be sufficient to override the motivational conflict of male mice in tasks with frequently shifting targets.

Even though a recent meta-analysis (Varholick et al., 2020) failed to find a consistent impact of social hierarchy on performance of male mice in traditional learning tests, stress due to excessive intermale aggression could reduce health status and indirectly affect performance in behavioral tests (Van Loo et al., 2003; Carvalheiro et al., 2021; Wikanthi et al., 2024; Zhu et al., 2024). We do not think this was a relevant factor in our study because no excessive intermale aggression was observed during inspection of the experiments and no bite wounds or changes in weight gain were found during health checks, neither before nor after the time point at which task motivation and performance of males in the dual motivation group collapsed. The enriched environment of the IntelliCage (Van Loo et al., 2003) and the cage cleaning schedule which avoided complete removal of scent marks (Hurst et al., 1993) may have reduced aggressive behavior and supported social tolerance.

Our study has several limitations. Despite the study’s emphasis on sex differences, female estrous cycle was not monitored because we considered this inappropriate in an experimental environment designed explicitly to reduce handling of the animals as far as possible. We also wanted to avoid a source of potential sex bias. For technical reasons, we could not directly measure fluid intake volumes of the mice but had to use lick numbers as a proxy which may differ by microstructure of drinking. We can thus not exclude minor effects of quinine exposure on fluid intake volume. We tested mice of one inbred strain at one particular age in one laboratory environment using a selected class of behavioral tests. Whether our conclusions can be generalized to other strains, ages, laboratory environments and test protocols remains to be tested by follow up studies.

Neither sex nor content of the joker side bottles and thus experimental group could be blinded. However, we do not consider this a serious risk of bias because data collection in IntelliCage is fully automated and independent of the experimenter. Body weight was measured using an automated digital balance with minimal risk of reading bias. Due to technical limitations we had to design our study such that group and sex factors were nested inside cages. Although we are confident that we could visually exclude confounding cage effects based on scatter plots of data from individual cages, only an independent replication of the experiment can provide final proof that the dual motivation effects on male mice which we claim based on our data and for which there is no prior evidence are not confounded by spurious cage effects. Finally, one mouse had to be removed from the experiment because of insufficient licking. Another mouse was excluded from the analysis of learning tasks because it refused to drink saccharin during initial adaptation. In view of the magnitude of the observed effects and the overall number of study subjects, these single exclusions are unlikely to have biased our conclusions.

In conclusion, our study demonstrates that by using dual motivation protocols based on a combination of incentive and disincentive taste stimuli, it is possible to create a set of spatial learning tasks for IntelliCage which implement 3R principles better by not enforcing learning by water restriction and at the same time meet the requirement of allowing mice of both sexes to learn. We found no evidence for quinine as disincentive stimulus causing obvious adverse effects on health, body weight or lick numbers as estimate of fluid intake. In addition, the observation that male mice were much less motivated than females to engage in learning tasks with frequently shifting targets revealed a sex difference in motivated behavior which is not apparent in protocols that enforce learning by water restriction.

## Data Availability

The raw data supporting the conclusions of this article will be made available by the authors, without undue reservation.

## Supplementary material

Supplementary Figure 1 Development of body weight for dual (Dual) and single motivation (Control) groups, along with data of two welfare control groups. One group (Neutral) which remained in IntelliCage on the free adaptation protocol in all phases and a group (T3) housed in standard type III cages throughout the experiment. Body weight was measured during the pre-test period (pre I/II), during the first nose poke adaptation phase (NPA I), and during post-learning recovery phases with nose poke adaptation (NPA IV, V, VI, VII). Body weight increased robustly in all groups during the study period. While no difference was evident between the dual motivation and control group, a clear sex effect was present with males weighing much more (time bin: F1,374 = 1678, *p* < 0.0001, ω^2^ = 0.60 + 1.538; group: F1,59 = 1.197, ns; group × time bin: F1,374 = 0.0685, ns; sex: F1,59 = 157.3, *p* < 0.0001, ω^2^ = 0.72 + 6.569; sex × time bin: F1,374 = 10.36, *p* = 0.0014, ω^2^ = 0.01; sex × group: F1,59 = 0.2726, ns; sex × group × time bin: F1,374 = 0.0000, ns). Considering all groups, the mice of the T3 group and, in males, the mice of the neutral group showed a slightly greater overall weight gain compared to the dual motivation and control groups (group: F3,87 = 5.806, *p* = 0.0012, ω^2^ = 0.14; group × time bin: F3,562 = 19.69, *p* < 0.0001, ω^2^ = 0.03; sex × group: F3,87 = 0.8468, ns; sex × group × time bin: F3,562 = 4.035, *p* = 0.0074, ω^2^ = .00).
