# Supplementary figures and images for: IntelliCage protocols using incentive-disincentive dual motivation work with male as well as female mice and reveal sex differences in motivated behavior
Source: Front Behav Neurosci. 2026 May 26;20:1833013. doi: 10.3389/fnbeh.2026.1833013 (PMC13246642; doi:10.3389/fnbeh.2026.1833013)

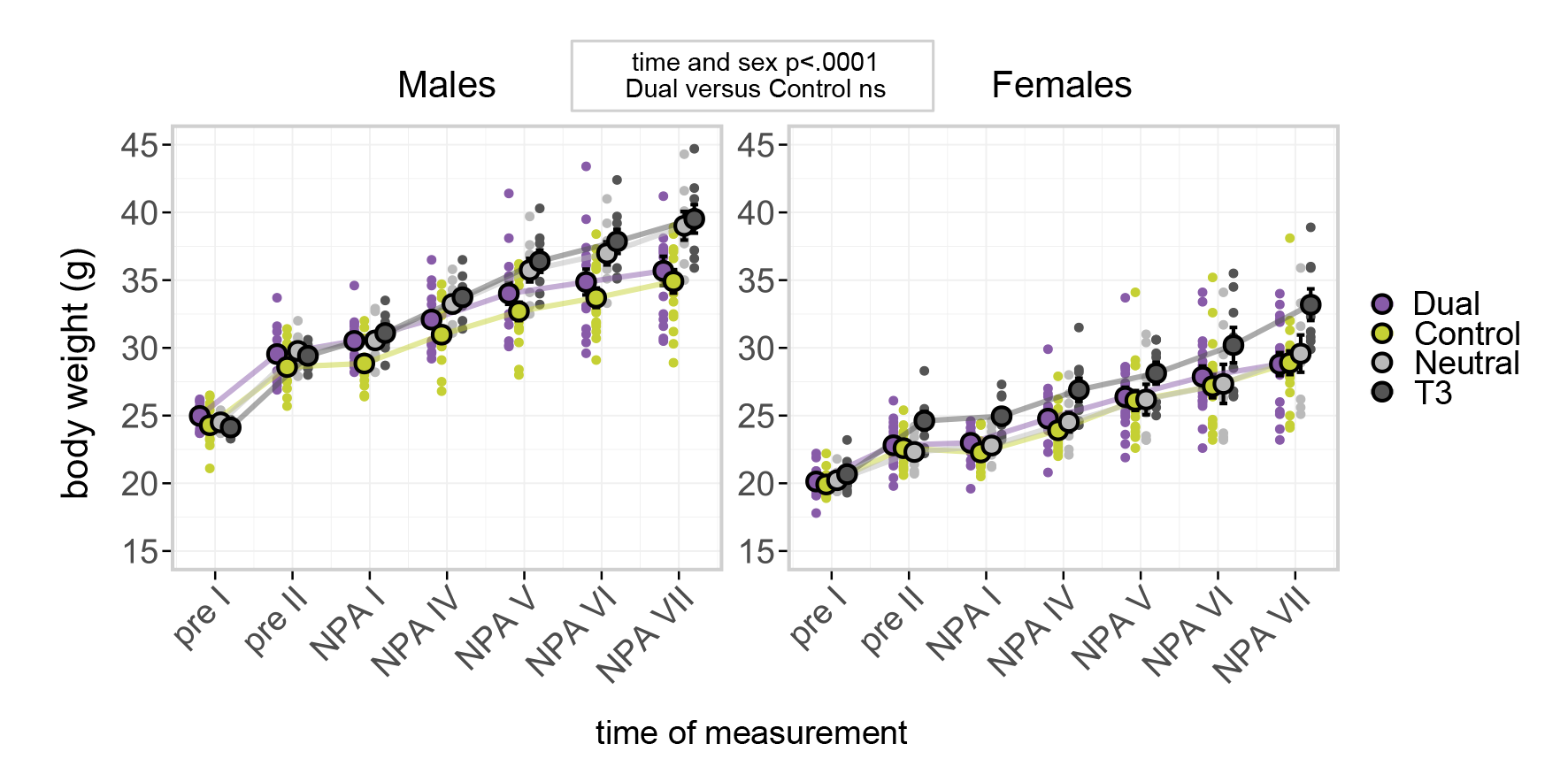

Supplement: Supplementary Figure 1 — Development of body weight for dual (Dual) and single motivation (Control) groups, along with data of two welfare control groups. One group (Neutral) which remained in IntelliCage on the free adaptation protocol in all phases and a group (T3) housed in standard type III cages throughout the experiment. Body weight was measured during the pre-test period (pre I/II), during the first nose poke adaptation phase (NPA I), and during post-learning recovery phases with nose poke adaptation (NPA IV, V, VI, VII). Body weight increased robustly in all groups during the study period. While no difference was evident between the dual motivation and control group, a clear sex effect was present with males weighing much more (time bin: F1,374 = 1678, p < 0.0001, ω2 = 0.60 + 1.538; group: F1,59 = 1.197, ns; group × time bin: F1,374 = 0.0685, ns; sex: F1,59 = 157.3, p < 0.0001, ω2 = 0.72 + 6.569; sex × time bin: F1,374 = 10.36, p = 0.0014, ω2 = 0.01; sex × group: F1,59 = 0.2726, ns; sex × group × time bin: F1,374 = 0.0000, ns). Considering all groups, the mice of the T3 group and, in males, the mice of the neutral group showed a slightly greater overall weight gain compared to the dual motivation and control groups (group: F3,87 = 5.806, p = 0.0012, ω2 = 0.14; group × time bin: F3,562 = 19.69, p < 0.0001, ω2 = 0.03; sex × group: F3,87 = 0.8468, ns; sex × group × time bin: F3,562 = 4.035, p = 0.0074, ω2 = .00). [file Image_1.tif]
